# Transcriptome analysis in different rice cultivars provides novel insights into desiccation and salinity stress responses

**DOI:** 10.1038/srep23719

**Published:** 2016-03-31

**Authors:** Rama Shankar, Annapurna Bhattacharjee, Mukesh Jain

**Affiliations:** 1 Functional and Applied Genomics Laboratory, National Institute of Plant Genome Research (NIPGR), Aruna Asaf Ali Marg, New Delhi - 110067, India; 2 School of Computational and Integrative Sciences, Jawaharlal Nehru University, New Delhi-110067, India

## Abstract

Drought and salinity are the major environmental factors that affect rice productivity. Comparative transcriptome analysis between tolerant and sensitive rice cultivars can provide insights into the regulatory mechanisms involved in these stress responses. In this study, the comparison of transcriptomes of a drought-tolerant [Nagina 22 (N22)] and a salinity-tolerant (Pokkali) rice cultivar with IR64 (susceptible cultivar) revealed variable transcriptional responses under control and stress conditions. A total of 801 and 507 transcripts were exclusively differentially expressed in N22 and Pokkali rice cultivars, respectively, under stress conditions. Gene ontology analysis suggested the enrichment of transcripts involved in response to abiotic stress and regulation of gene expression in stress-tolerant rice cultivars. A larger number of transcripts encoding for members of NAC and DBP transcription factor (TF) families in N22 and members of bHLH and C_2_H_2_ TF families in Pokkali exhibited differential regulation under desiccation and salinity stresses, respectively. Transcripts encoding for thioredoxin and involved in phenylpropanoid metabolism were up-regulated in N22, whereas transcripts involved in wax and terpenoid metabolism were up-regulated in Pokkali. Overall, common and cultivar-specific stress-responsive transcripts identified in this study can serve as a helpful resource to explore novel candidate genes for abiotic stress tolerance in rice.

Rice is one of the most important staple food crop consumed by more than half of the world population. The adverse environmental conditions, mainly drought and salinity, impose major challenges to rice productivity^1^. Both these stresses can affect the plants together too, because excessive evaporation due to drought stress leads to salt accumulation in soil. Several genes have been reported to modulate drought and salinity stress responses in rice^2^,^3^. The effects of these stresses vary at different stages of development. Particularly, seedling and reproductive stages are more prone to stress as compared to tillering and grain filling stages in rice^4^.

A large number of stress-tolerant rice cultivars/landraces have been identified based on their phenotypic and physiological responses under various abiotic stress conditions^4^,^5^,^6^,^7^. Microarray analyses have been carried out to identify the molecular level variations among the stress-sensitive and -tolerant rice cultivars^8^,^9^,^10^. These studies have revealed a higher number of differentially regulated genes in stress-sensitive as compared to stress-tolerant rice cultivars under stress conditions^8^,^9^. A large number of transcription factors (TFs) belonging to different families have been reported to be involved in stress-response pathways^11^,^12^,^13^. Various catalytic, DNA binding, transcription regulator activities, and carbon fixation, glycolysis and α-linolenic metabolic pathways have also been found to be enriched in stress-tolerant rice cultivars^8^. Many genes encoding for enzymes involved in accumulation of osmolytes, reactive oxygen species (ROS), ion transportation and signaling pathways have been selected as promising targets to engineer abiotic stress tolerance in economically important crops^14^,^15^. However, sufficient progress has not been made towards generation of stress-tolerant rice as of now. Therefore, it is important to gain in-depth knowledge about the complexity of transcriptional regulation in rice during drought and salinity stresses using advanced technologies.

The high accuracy and sensitivity of RNA-seq makes it one of the most suitable techniques to study the whole transcriptome^16^,^17^. It allows identification of alternative splicing (AS) events, novel genes/transcripts and digital gene expression at the isoform level as compared to microarray and expressed sequence tag sequencing^16^,^18^,^19^,^20^. Recently, RNA-seq has been used to explore the transcriptomes of several plants under abiotic stress conditions^21^,^22^,^23^,^24^,^25^.

In this study, we performed RNA-seq analysis to explore the transcriptional variations among three rice cultivars, including IR64 [stress-sensitive (SS)], Nagina 22[(N22), drought-tolerant (DT)] and Pokkali [salinity-tolerant (ST)] under control and stress conditions. The transcriptomes of IR64, N22 and Pokkali rice cultivars under control, desiccation and salinity stress conditions at the seedling stage were sequenced and various stress-responsive novel genes/transcript isoforms were identified. The differential gene expression patterns and variations in AS events in response to abiotic stresses in the three rice cultivars were also analyzed. Functional categorization of differentially expressed transcripts was carried out to reveal various metabolic pathways involved in stress responses. Overall, this study provides a comprehensive overview of transcriptional regulation and complexity in stress-sensitive and stress-tolerant rice cultivars under desiccation and salinity stresses.

## Results

### Discovery of novel transcripts through transcriptome sequencing

Total mRNA from seedlings of three rice cultivars, IR64 [high yielding, but susceptible to abiotic stresses^4^], Nagina 22 (N22) [drought-tolerant^26^] and Pokkali [salinity-tolerant^27^], were sequenced under control, desiccation and salinity stress conditions using Illumina system. In total, we obtained more than 180 million raw reads for each rice cultivar with at least 60 million reads for each condition (control, desiccation and salinity) (Table 1). These reads were filtered using NGS QC toolkit^28^ to remove the low-quality reads. More than 84% of high quality (HQ) reads (Table 1) with average Phred quality score of ≥30 at each base position (Supplementary Fig. S1) were obtained and used for downstream analyses. Reference-based transcriptome assemblies of IR64, N22 and Pokkali RNA-seq data were performed employing TopHat-Cufflinks pipeline using the rice genome sequence as reference. More than 93% of HQ reads from individual sample (control, desiccation and salinity) of each cultivar (IR64, N22 and Pokkali) could be mapped on the rice genome. The assembly of mapped reads resulted in the identification of a total of 88652, 90556 and 87887 transcripts in IR64, N22 and Pokkali rice cultivars, respectively (Table 1). A summary of the assembly statistics has been provided in Supplementary Table S1.

**Figure 1:**
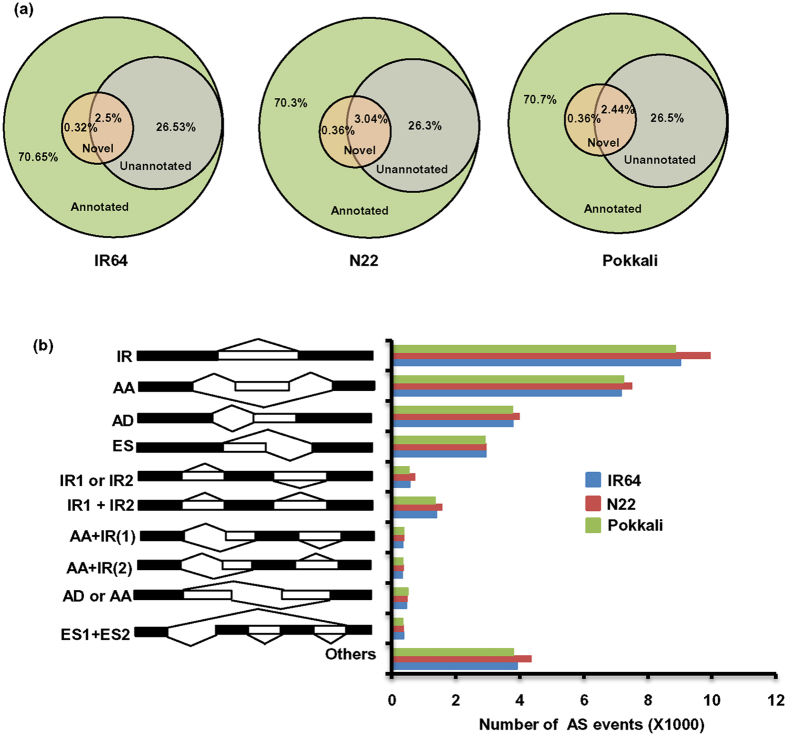
Annotation and alternative splicing (AS) events in the transcriptomes of IR64, N22 and Pokkali rice cultivars. (**a**) Categorization of all the transcripts into annotated, un-annotated, and novel transcripts (annotated and un-annotated) in IR64, N22 and Pokkali rice cultivars. (**b**) Distribution of various AS events in IR64, N22 and Pokkali rice cultivars is shown. IR-intron retention, AA-alternate-3′-acceptor, AD-alternate-5′-donor and ES-exon skipping.

Identification of novel genes/transcript isoforms has emerged as one of the major advantages of RNA-seq analysis^16^,^17^. We identified a total of 17444, 18424 and 16963 novel transcript isoforms in IR64, N22 and Pokkali rice cultivars, respectively. In addition, a total of 2499 (1832 loci), 3054 (2265 loci) and 2433 (1785 loci) novel transcripts in IR64, N22 and Pokkali, respectively, could also be identified. The average length of novel transcripts was found to be lesser (1070 bp in IR64, 1111 bp in N22 and 1028 bp in Pokkali) than that of known (annotated) transcripts for each rice cultivar (Supplementary Fig. S2) similar to that reported in previous studies^18^,^19^,^29^. More than 70% of the assembled transcripts for each cultivar were assigned with a putative function. A total of 10.6%, 11.9% and 14.5% of novel transcripts (0.3%, 0.4% and 0.4% of total transcripts) in IR64, N22 and Pokkali rice cultivars, respectively, could also be assigned with a putative function (Fig. 1a). Although the identified novel transcripts/transcript isoforms will be validated in future experiments, they were included in further analyses for preliminary functional characterization and investigating their putative role in abiotic stress responses.

GOSlim term analysis revealed that genes involved in biological process (≥14%) followed by protein modifications (≥5%), defense response (≥4%), response to abiotic stress (≥4%), translation (≥3%), DNA repair (≥3%) and regulation of transcription (≥3%) represented about 40% of the novel transcripts, whereas the remaining 60% transcripts were found to be involved in other processes (Supplementary Fig. S3). In the molecular function category, >40% of the novel transcripts were involved in ATP binding, DNA binding, protein binding and metal ion binding in all the three rice cultivars and the rest of the novel transcripts were found to be involved in catalytic activity, GTPase activity, NADH dehydrogenase activity, oxidoreductase activity, transferase activity, protein transporter activity and ion transporter activity. Further, a total of 65% of novel transcripts belonged to nucleus, membrane, chloroplast, mitochondria and cytosol, cellular component GOSlim categories in all the three rice cultivars (Supplementary Fig. S3). Interestingly, some reports have highlighted that genes encoding for proteins residing in the nucleus and involved in DNA/nucleotide binding and signal transduction pathways can function as TFs^30^,^31^, which can regulate stress response/adaptation.

The eukaryotic orthologous group (KOG) analysis revealed that more than 42% of the novel transcripts were involved in various functions, mainly lipid transport and metabolism (7%), intracellular trafficking (≥7%), post-translational modifications (≥8%), translation (6%), amino acid transport and metabolism (5%), signal transduction mechanism (≥5%) followed by general functions (≥4%) in all the rice cultivars (Supplementary Fig. S4). The number of transcripts involved in general functions was higher in IR64 as compared to others. However, the transcripts involved in lipid transport and metabolism were more in N22 and Pokkali cultivars, whereas the transcripts involved in signal transduction mechanisms were greater in N22 cultivar (Supplementary Fig. S4).

### AS events in rice cultivars

AS results in production of various transcript isoforms from a single gene and enhances the transcriptional complexity and diversity. Our analysis revealed that intron retention (IR) is the most dominant AS event represented by 9016, 9962 and 8852 events in IR64, N22 and Pokkali cultivars, respectively (Fig. 1b). Other major AS events were identified as alternate 3′ acceptor (AA) (7164 in IR64, 7511 in N22 and 7238 in Pokkali), alternate 5′ donor (AD) (3788 in IR64, 3992 in N22 and 3769 in Pokkali) and exon skipping (ES) (2941 in IR64, 2953 in N22 and 2918 in Pokkali). Among the mixed-type AS events, the IR1 + IR2 type events were highest in number (1403 in IR64, 1581 in N22 and 1351 in Pokkali). However, ES1 + ES2 events were least frequent in all the rice cultivars analyzed (Fig. 1b). Overall, the frequency of AS events was higher in N22 as compared to IR64 and Pokkali rice cultivars.

The frequency of different AS events under control and stress conditions within each rice cultivar was found to be similar to the AS events predicted at the whole transcriptome level (Supplementary Table S2). Interestingly, the frequency of total number of AS events was lesser under stress as compared to control condition in all the rice cultivars. IR represented the major AS events (~40%) under all the conditions in each rice cultivar. However, a reduced frequency of IR AS events was observed in IR64 and N22 under stress conditions as compared to control, with the exception of Pokkali cultivar (Supplementary Table S2). Similar observations have been made under salinity stress in a previous study also^32^. However, the frequency of ES and ES1 + ES2 events increased under stress conditions in all rice cultivars. Further investigations are needed to unfurl the basis of such differential occurrences of AS events in the rice cultivars.

Functional categorization of the transcripts produced through IR AS event revealed their involvement in various pathways related to stress responses. Transcripts related to carbohydrate metabolic process and response to abiotic stress were overrepresented in N22 under desiccation stress conditions. However, transcripts involved in transport, nucleic acid metabolic processes, localization and cellular response to stimulus were enriched in Pokkali under salinity stress (Supplementary Fig. S5).

### Differential gene expression among contrasting rice cultivars

To study transcriptional variations among different rice cultivars under control and stress (desiccation and salinity) conditions, differential gene expression analysis was performed. Overall, the transcriptomes of each cultivar under control and stress conditions were more similar to each other as compared to other cultivars. The correlation between transcriptomes of IR64 under control and stress conditions was less as compared to that of N22 and Pokkali (Supplementary Fig. S6).

**Figure 2:**
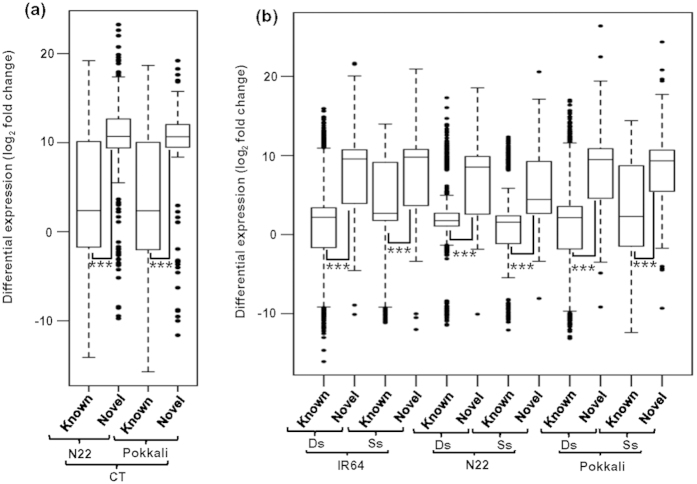
Differential gene expression of known and novel transcripts in IR64, N22 and Pokkali rice cultivars. (**a**) Boxplot showing distribution of differential gene expression (log_2_ fold change) of novel and known transcripts under control condition (CT) in N22 and Pokkali rice cultivars and (**b**) under stress [desiccation stress (Ds) and salinity stress (Ss)] conditions in IR64, N22 and Pokkali rice cultivars. Significant differences (*P* ≤ 1.43e-08) are marked with asterisks.

The analysis revealed a total of 3119 and 756 significantly differentially expressed transcripts in IR64 cultivar under desiccation and salinity stress, respectively. Similarly, a total of 2510 and 1809 transcripts in N22, and 3132 and 1036 transcripts in Pokkali were differentially expressed under desiccation and salinity stresses, respectively. In addition, we identified a total of 859 and 587 differentially expressed transcripts in N22 and Pokkali, respectively, as compared to IR64 under control condition (Supplementary Table S3). A comparative analysis of differential expression patterns of known and novel transcripts was carried out. Overall, the novel transcripts were differentially expressed at higher level as compared to the known transcripts (*P*-value ≤ 1.43e-08) in all the rice cultivars under control (Fig. 2a) and stress conditions (Fig. 2b).

**Figure 3:**
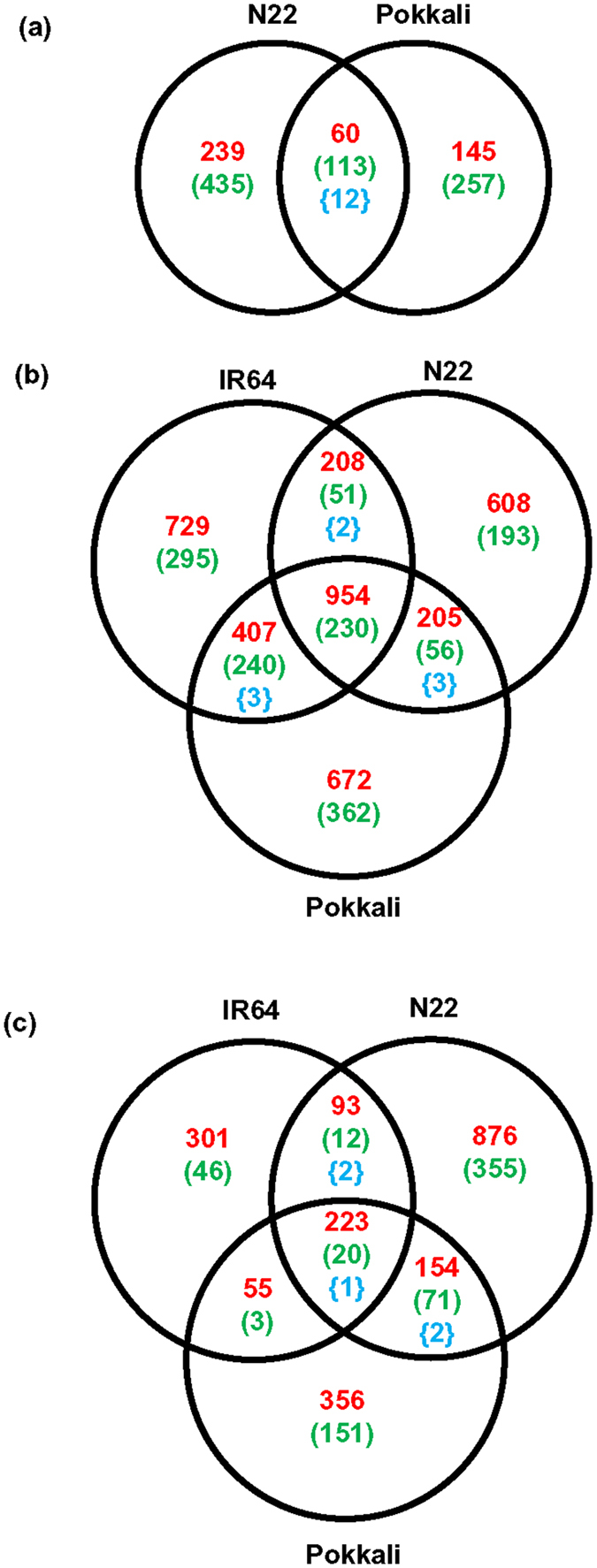
Common and specific differentially regulated transcripts in IR64, N22 and Pokkali rice cultivars under control and stress conditions. (**a**) Differentially up-regulated (in red color), down-regulated (in green color) and transcripts up-regulated/down-regulated (in blue color) in N22 and Pokkali rice cultivars with respect to IR64 cultivar under control condition are shown. (**b**,**c**) Differentially regulated transcripts under desiccation (**b**) and salinity stresses (**c**) in IR64, N22 and Pokkali rice cultivars as compared to the control condition are shown.

Comparative transcriptome analysis among the rice cultivars revealed that a total of 60 up-regulated and 113 down-regulated transcripts were common in both N22 and Pokkali under control condition (Fig. 3a). However, a total of 674 and 402 transcripts were differentially regulated specifically in N22 and Pokkali cultivars, respectively, under control condition. Further, a total of 954 and 230 transcripts were found to be commonly up-regulated and down-regulated, respectively, in all the three rice cultivars under desiccation stress (Fig. 3b). In addition, 259 transcripts in IR64/N22, 647 in IR64/Pokkali and 261 in N22/Pokkali were commonly regulated under desiccation stress. However, a total of 1024, 801 and 1034 transcripts were differentially expressed specifically in IR64, N22 and Pokkali, respectively, under desiccation stress (Fig. 3b). The comparison of differential expression profile of transcripts among the three rice cultivars under salinity stress revealed a total of 223 up-regulated and 20 down-regulated transcripts common among all the three rice cultivars (Fig. 3c). In addition, a total of 105, 58 and 225 transcripts were commonly regulated in IR64/N22, IR64/Pokkali and N22/Pokkali, respectively. Notably, a total of 347, 1231 and 507 transcripts were found to be differentially expressed specifically in IR64, N22 and Pokkali rice cultivars, respectively, under salinity stress (Fig. 3c).

### Differential expression of alternatively spliced transcripts

**Figure 4:**
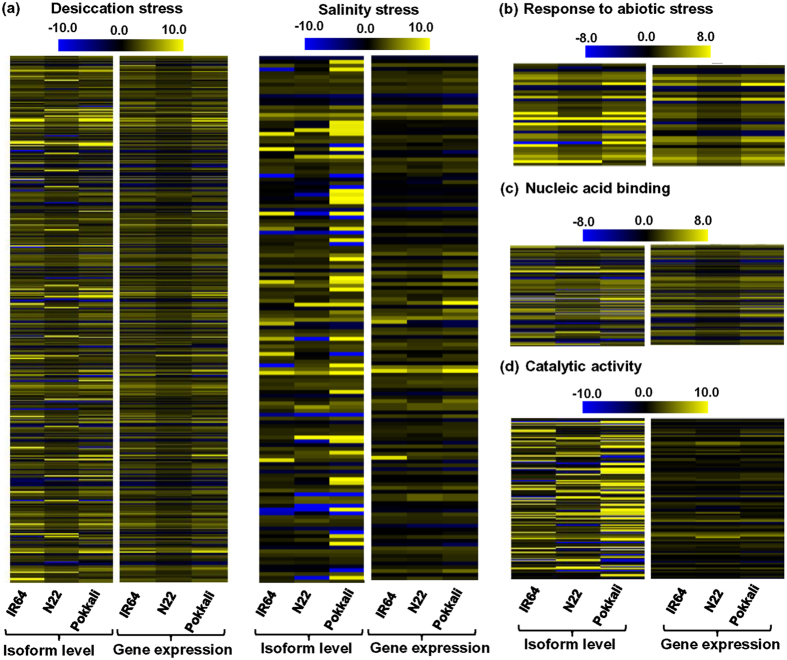
Differential expression of transcripts produced through intron retention (IR) event in the rice cultivars. (**a**) Heatmaps showing the expression profiles of IR isoforms and genes regulated by desiccation and salinity stresses in IR64, N22 and Pokkali. (**b–d**) Expression profiles of IR isoforms involved in response to abiotic stress and (**b**) nucleic acid binding under desiccation stress (**c**), and catalytic activity (**d**) under salinity stress are shown. Color scale represents log_2_ fold change values.

A large number of the transcripts generated as a result of AS were found to be differentially expressed in the rice cultivars under desiccation and/or salinity stresses (Supplementary Table S4). Intron retention (IR) was the dominant AS event; hence the expression profiles of IR transcripts were compared at isoform and gene levels. The IR transcript isoforms showed higher variations in the differential expression pattern as compared to gene level under desiccation and salinity stresses (Fig. 4a). The greater differential expression range at isoform level can give better evidence about the regulation of novel/less-explored transcripts participating in stress response pathways. Functional categorization of the differentially expressed IR transcripts depicted that glycoside metabolic process, disaccharide metabolic process, trehalose metabolic process and catalytic activity terms were significantly enriched under desiccation and salinity stresses in all the rice cultivars (Table 2). However, gene ontology (GO) terms related to response to abiotic stimulus, response to desiccation stress, catabolic process, nucleic acid binding and ATPase activity were found to be significantly enriched under desiccation stress, and transcripts involved in carbohydrate metabolic process, di- and tri-valent cation, and calcium ion transport were enriched under salinity stress in the rice cultivars (Table 2). Variation in expression patterns at gene level was found to be less as compared to the expression patterns at isoform level for these terms (Fig. 4). Representatively, we compared the expression patterns at gene and isoform levels for genes related to response to abiotic stress (Fig. 4b), nucleic acid binding (Fig. 4c) and catalytic activity GO terms (Fig. 4d), under stress conditions. The expression variations at gene level (log_2_ fold change) were significantly lesser as compared to differential expression level (log_2_ fold change) at transcript isoforms level for all the genes associated with these terms.

**Figure 5:**
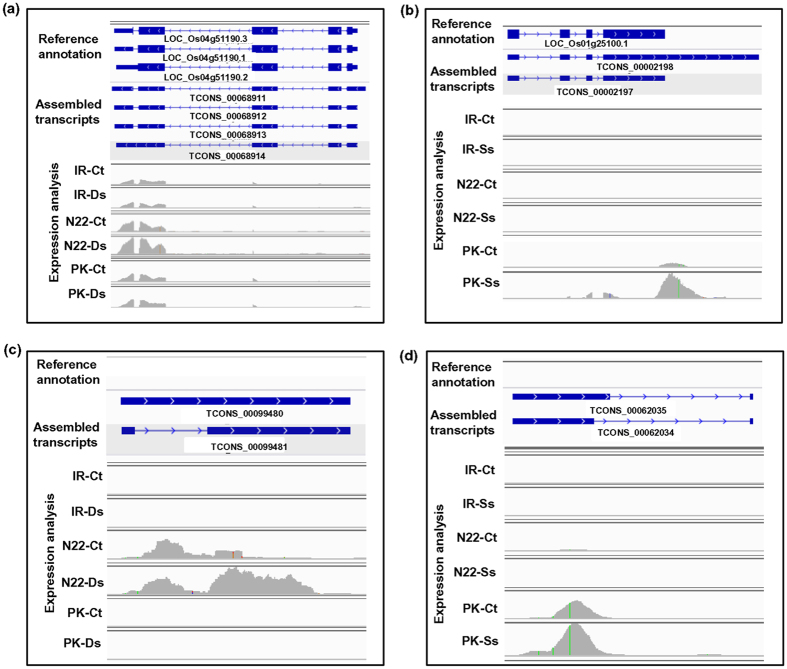
Expression patterns of transcript isoforms and novel transcripts specific to the rice cultivar(s) under control and/or stress conditions. (**a,c**) Integrative Genomics Viewer (IGV) visualization of representative transcript isoforms (**a**) and novel transcripts (**c**) in N22 rice cultivar showing higher expression under desiccation stress.(**b,d**) Representative transcript isoforms (**b**) and novel transcripts (**d**) in Pokkali rice cultivar showing higher expression under salinity stress. IR, IR64 rice cultivar, N22, Nagina 22 rice cultivar, PK, Pokkali rice cultivar, Ct- control, Ds- desiccation stress and Ss- salinity stress.

According to the previous study, the frequency and diversity of transcript isoforms can be genotype-specific and/or modulated by stress responses, resulting in accumulation of specific mRNAs^33^,^34^. We also observed genotype and/or stress-specific diversity of transcript isoforms in the rice cultivars. For example, a total of four transcript isoforms (TCONS_00068911, TCONS_000689128, TCONS_00068913 and TCONS_00068914) were identified for LOC_Os04g51190 (encoding for growth regulating factor) in our transcriptome data and their expression level was found to be significantly higher in N22 cultivar under desiccation stress as compared to any other genotype/condition (Fig. 5a). Similarly, two transcript isoforms (TCONS_00002197 and TCONS_00002198) for LOC_Os01g25100 (glutathione S-transferase) showed enhanced expression in Pokkali cultivar specifically under salinity stress (Fig. 5b). In addition, various novel transcripts, such as TCONS_00099480 and TCONS_00099481 were found to be specifically expressed only in N22 cultivar and were up-regulated under desiccation stress (Fig. 5c). Likewise, several novel transcripts, such as TCONS_00062034 and TCONS_00062035, were found to be specifically expressed in Pokkali cultivar only (Fig. 5d).

### Differential expression of transcription factors (TFs)

**Figure 6:**
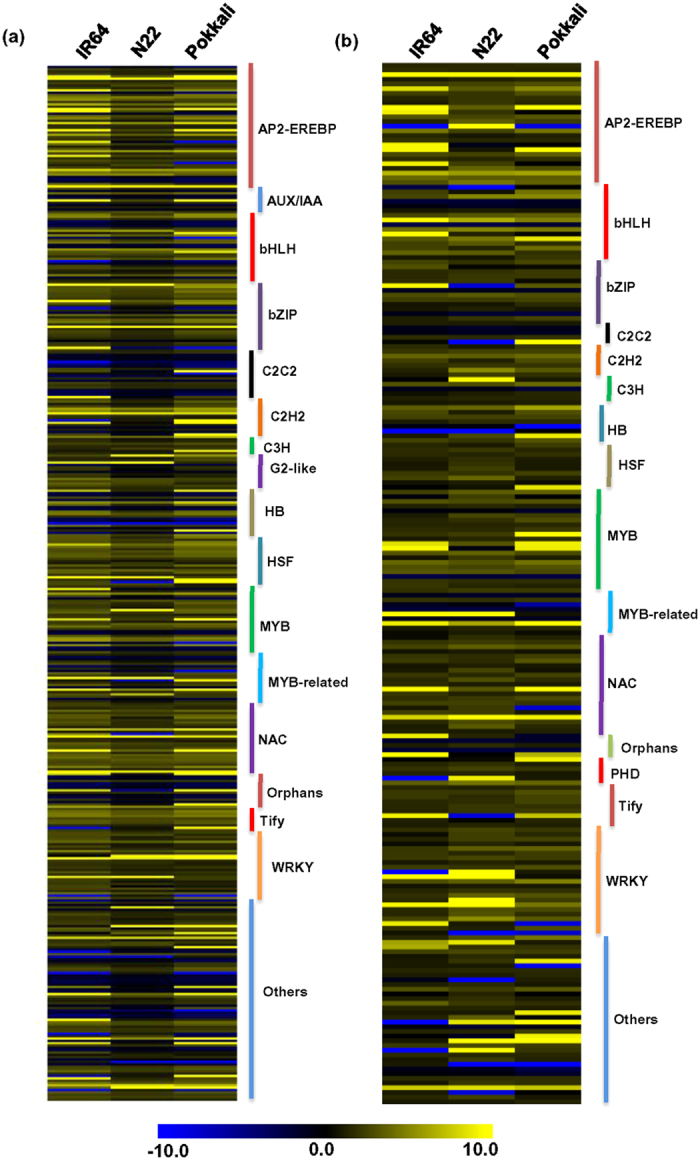
Transcription factor families differentially expressed in the rice cultivars under desiccation and salinity stresses. Heatmaps depicting the expression profile of differentially expressed TF families (mentioned on the right side) in IR64, N22 and Pokkali rice cultivars under desiccation (**a**) and salinity stresses (**b**) are shown. Color scale represents log_2_ fold change values.

Further, we analyzed differential expression of TF encoding transcripts in the rice cultivars under desiccation and salinity stresses. A total of 279, 261 and 270 transcripts encoding TFs were differentially expressed in IR64, N22 and Pokkali cultivars, respectively, under desiccation stress. Similarly, a total of 68, 173 and 101 transcripts with TF activities were differentially expressed in IR64, N22 and Pokkali cultivars, respectively, under salinity stress. Among the differentially expressed TFs, AP2/EREBP, bZIP, HB, MYB-related, WRKY and HSF families were found to be represented by more than 69% and 62% of the TF encoding transcripts commonly in N22 and Pokkali under desiccation and salinity stresses, respectively (Fig. 6a,b). Besides this, DBP and NAC TFs in N22 under desiccation stress^7^, and bHLH, C_2_H_2_ and MYB TFs in Pokkali under salinity stress were enriched. These TFs have already been reported to be involved in various abiotic stress response pathways in different plants^3^,^11^,^13^,^35^.

### Functional categorization of differentially expressed transcripts

**Figure 7:**
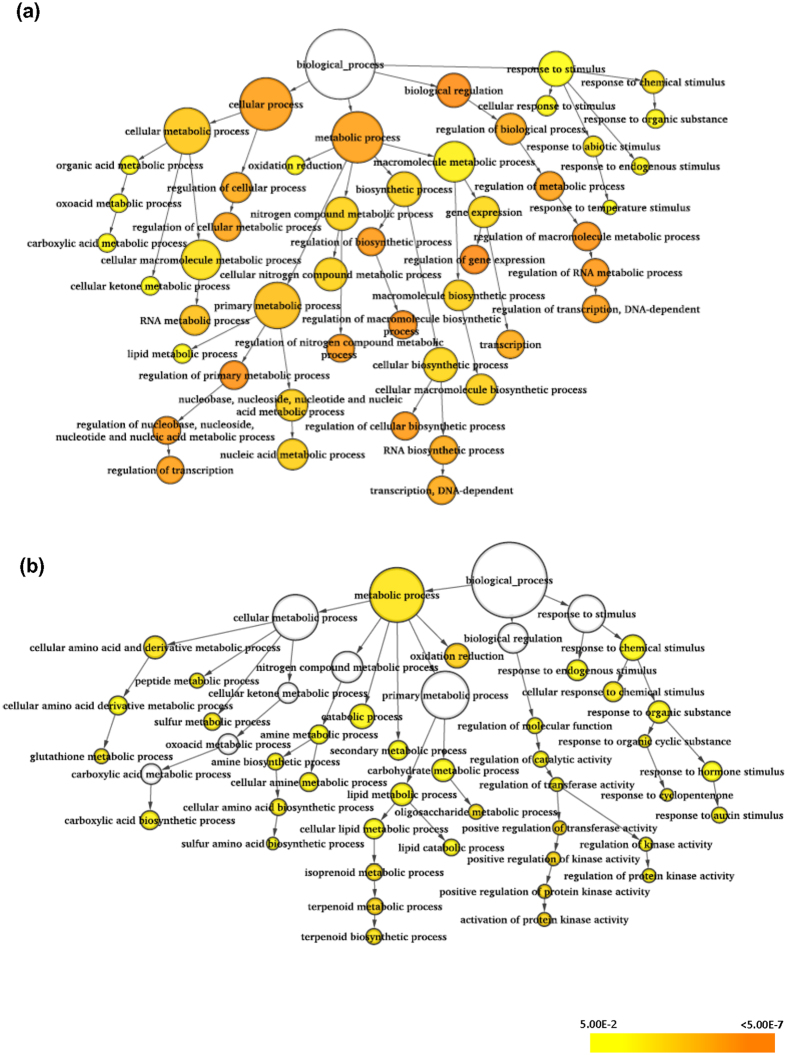
Gene ontology (GO) enrichment analysis of differentially expressed transcripts under stress in specific rice cultivars. GO enrichment for differentially expressed transcripts specific to N22 rice cultivar under desiccation stress (**a**) and specific to Pokkali rice cultivar under salinity stress (**b**) are shown. Node size is proportional to the number of transcripts in that category and color shaded is according to significant level (white represents-no significance difference, color scale yellow-*P*-value = 0.05, orange- *P*-value < 0.0000005).

GO enrichment analysis was performed for differentially regulated transcripts under control and stress conditions to gain more insights into their involvement in various biological processes under stress conditions in rice. The response to stimulus, terpenoid metabolic process and aromatic compound biosynthesis terms were found to be common among the differentially expressed transcripts in N22 and Pokkali rice cultivars under control condition (Supplementary Fig. S7a,b). Ion transport, cellular catabolic process and amino acid activation terms were specifically enriched in N22 (Supplementary Fig. S7a), whereas lipid metabolic process, cell growth and small molecule biosynthesis process terms were found to be specific to Pokkali cultivar (Supplementary Fig. S7b). In addition, differentially expressed transcripts specific to N22 and Pokkali under desiccation and salinity stress conditions, respectively, were subjected to GO enrichment analysis. GO terms, mainly cellular response to stimulus, response to endogenous stimulus, regulation of transcription and RNA metabolic processes were significantly enriched in N22 under desiccation stress (Fig. 7a), whereas oxidation reduction, response to auxin stimulus, terpenoid biosynthesis, glutathione-metabolic processes and activation of kinase activity, GO terms were enriched in Pokkali under salinity stress (Fig. 7b).

### Metabolic pathways under stress conditions

**Figure 8:**
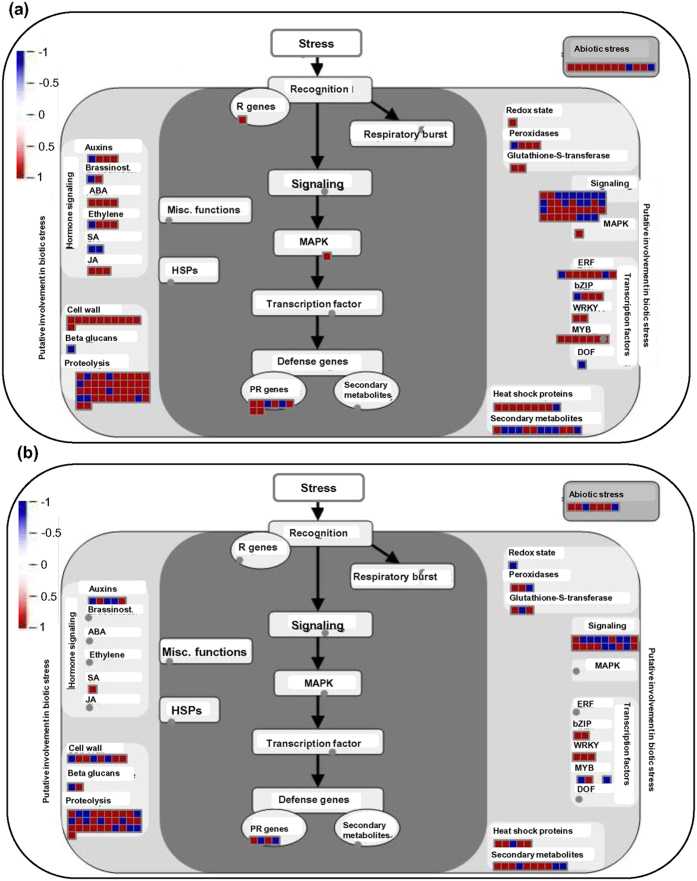
Overview of metabolic pathways involving differentially expressed transcripts in specific rice cultivars under stress conditions. Metabolic pathway overview (from Mapman) for the differentially expressed transcripts specific to N22 (**a**) and Pokkali (**b**) rice cultivars under desiccation and salinity stresses, respectively, are shown. Red, up-regulated transcripts and blue, down-regulated transcripts.

Differentially regulated transcripts specific to tolerant rice cultivars were further analyzed to investigate their involvement in various metabolic pathways using Mapman. The metabolic pathway overview represented the enrichment of secondary metabolite and starch biosynthesis, besides amino acid and cell wall biosynthetic pathways in N22 and Pokkali rice cultivars. However, genes involved in cell wall, secondary metabolites and lipid biosynthesis pathways were significantly enriched among the up-regulated genes in N22 under desiccation stress as compared to those in Pokkali under salinity stress (Supplementary Fig. S8). Similarly, cellular overview revealed that genes encoding for thioredoxin and involved in cold and drought stress were up-regulated in N22 under desiccation stress, whereas genes related to cell division and glutaredoxin were differentially expressed in Pokkali under salinity stress (Supplementary Fig. S9). The secondary metabolite pathway overview indicated that the genes involved in lignin, phenylpropanoid, flavonoid and dihydroflavonol metabolic pathways were significantly enriched in N22 under desiccation stress (Supplementary Fig. S10a), whereas genes involved in carotenoid, anthocyanin, wax and shikimate metabolic pathway were significantly enriched specifically in Pokkali under salinity stress (Supplementary Fig S10b). Further, the stress response pathways specific to N22 and Pokkali under desiccation and salinity stresses, respectively, were also studied. We observed that genes involved in signaling pathways, including components of MAPK, ABA, jasmonic acid, ethylene, ERF and stress-responsive transcription regulatory elements (including TFs) were enriched in N22 under desiccation stress (Fig. 8a). Similarly, genes involved in salicylic acid (SA) hormone signaling pathway, transcription regulation and glutathione-S-transferase were found to be enriched in Pokkali rice cultivar under salinity stress (Fig. 8b).

### Validation of differential gene expression

**Figure 9:**
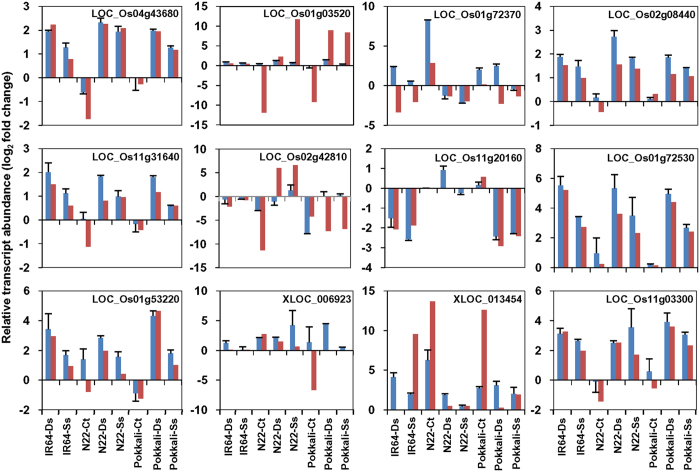
Validation of RNA-seq data *via* qRT-PCR. Bar graphs depicting the relative transcript abundance of selected transcripts in the rice cultivars under different conditions are shown. All the data points are represented by the log_2_ fold change values. The N22-Ct and Pokkali-Ct expression have been calculated using IR64 (control) as a reference and expression under stress (desiccation and salinity) conditions have been also calculated with respect to control condition for each cultivar. The blue bars represent the expression *via* qRT-PCR and red bars represent expression *via* RNA-seq. The identifiers with prefix XLOC represent novel transcripts. Ct-control condition, Ds-desiccation stress and Ss-salinity stress.

The differential expression results obtained from RNA-seq were validated by quantitative reverse transcription PCR (qRT-PCR) analysis. A total of 14 transcripts were selected randomly for qRT-PCR analysis. The qRT-PCR was performed under all the three conditions (control, desiccation and salinity) for each rice cultivar. The Ct values obtained were normalized against the internal control and fold change was calculated under desiccation and salinity stresses in all three rice cultivars. In addition, fold change of transcripts in N22 and Pokkali with respect to IR64 under control condition were also calculated. The differential expression values of all the selected transcripts obtained by qRT-PCR analysis were plotted along with the RNA-seq data. We observed similar differential expression patterns (correlation coefficient of 0.72) (Supplementary Fig. S11) of the transcripts in qRT-PCR and RNA-seq data analyses (Fig. 9).

## Discussion

Various environmental stresses, mainly drought and salinity adversely affect rice production. Over the years, several stress-tolerant rice landraces/cultivars have been identified, which can withstand the onslaught of environmental perturbations^9^,^36^,^37^. However, most of these stress-tolerant rice cultivars are low-yielding, in contrast to stress-susceptible but high-yielding cultivars^38^,^39^,^40^. To develop a better understanding of the molecular level variations among the stress-sensitive and -tolerant rice cultivars, we sequenced the whole transcriptome of three rice cultivars with contrasting stress responses under control and stress conditions. The transcriptome assembly led to the discovery of a large number of novel transcripts and transcript isoforms in the three rice cultivars. Many of them exhibited stress-responsive gene expression. A greater percentage (50%) of AS events could be identified in our analysis as compared to previous reports in rice and Arabidopsis^18^,^20^. This implies that RNA-seq technology coupled with improved strategy of analysis enabled recognition of novel transcript isoforms and allowed us to gain deeper insights into their putative roles in abiotic stress responses.

Recent studies have reported the prevalence of AS events in plants and their roles in crucial regulatory processes^41^,^42^. IR has been recognized as the most dominant AS event in plants^43^,^44^. We also found that majority of transcripts identified in the three rice cultivars were generated due to IR event. The AS events often lead to production of truncated proteins in animals and plants^45^,^46^. Such truncated proteins have been implicated in jasmonate signaling, photosynthetic processes and energy production in plants^44^,^47^,^48^. Interestingly, several proteins arising from specific AS transcripts have also been reported to be related to heat and salinity stress responses in plants^18^,^32^. Notably, we observed a decrease in most of AS events under desiccation stress in all the rice cultivars. The decrease in the frequency of AS events may be a strategy employed by the rice cultivars towards energy conservation as a stress adaptive mechanism. However, the occurrence of AS events was found to be greater in Pokkali under salinity stress, which is consistent with a previous report in Arabidopsis^32^. The existence of greater number of AS variants in Pokkali under salinity stress condition possibly indicates the occurrence of a different mechanism for stress adaptation. These evidences imply that AS events play a crucial role in regulating gene expression under various stress conditions in plants.

Previously, it was reported that differential expression analysis at isoform level can provide us more comprehensive overview of gene regulation as compared to gene level expression patterns^49^,^50^. Therefore, we analyzed the differential expression profile at transcript isoform level to obtain stress-responsive signatures in the rice cultivars. The expression levels of transcript isoforms showed greater variation as compared to their respective gene level in the rice cultivars under stress conditions. More than 21% of the differentially expressed transcripts in N22/IR64 and Pokkali/IR64 under control condition were common, suggesting the involvement of transcripts with overlapping and/or specific roles in drought and salinity stress responses. In an earlier study, a salt-sensitive rice genotype exhibited differential regulation of a larger number of transcripts as compared to the tolerant genotype under salinity stress^8^. Likewise, we also found a greater number of differentially regulated transcripts in IR64 as compared to N22 under desiccation stress. However, a lesser number of transcripts were differentially expressed in IR64 as compared to Pokkali under salinity stress. This could be due to genotype-specific response, method of stress implementation or strategy employed for transcriptome analysis (RNA-seq) in our study versus microarray in the previous studies^7^,^8^,^9^.

Stress-responsive TFs have been considered as master regulators in abiotic stress responses in plants^51^,^52^. In corroboration with previous studies, we also found various stress-responsive TFs, like AP2/EREBP, NAC, bHLH, HB, MYB, MYB-related, WRKY, C_2_H_2_, and bZIP to be enriched in N22 and Pokkali cultivars under stress conditions^7^,^8^,^19^,^34^. This suggests the possible involvement of these TFs in conferring stress-adaptive traits to the tolerant rice cultivars. The enrichment of genes involved in cellular response to stimulus, oxidation reduction and regulation of gene expression in N22, and glutathione metabolic process, terpenoid biosynthesis and auxin stimulus in Pokkali under stress conditions, further signified their involvement in modulation of various physiological responses crucial during stress responses in rice, as reported earlier also^8^,^53^,^54^.

The role of plant hormones in abiotic stress responses is also well documented^55^,^56^. The genes participating in several hormone signaling pathways, like ABA, SA, ethylene and jasmonic acid (JA) were found to be enriched in N22 under desiccation stress. However, genes involved in auxin biosynthesis were found to be enriched in Pokkali rice cultivar under stress condition similar to previous reports^55^,^56^. Further, the role of MAP kinases (MAPK) in abiotic stress responses in plants has been delineated^57^,^58^. The overexpression of few MAPK genes was found to confer stress tolerance in transgenics^59^,^60^. Interestingly, we also obtained the enrichment of genes involved in MAPK signaling pathways in N22 under desiccation stress. This suggests the involvement of MAPK signaling pathways in stress responses in the rice cultivars.

Cell wall undergoes extensive remodeling in plants to elicit an adaptive mechanism for survival under stress conditions^61^,^62^. It has been found that many proteins help to maintain the osmotic potential inside the cell during stress^63^. Our study revealed the enrichment of genes involved in cell wall maintenance in N22 and Pokkali rice cultivars, which possibly contributes to their drought and salinity tolerant phenotype, respectively. Particularly, wax being the major component of cell wall outer cuticle layer, inhibits the excess water evaporation from leaves, which acts as a possible adaptive mechanism in plants to evade the detrimental effects of salinity stress. Recently, the overexpression of genes involved in wax biosynthesis has led to development of salinity tolerance in crop plants^64^,^65^. Interestingly, we also found that genes involved in wax metabolism were up-regulated in Pokkali rice cultivar. These genes may contribute to the salinity tolerance of Pokkali rice cultivar. In addition, the role of lignin in cell wall maintenance in plants during abiotic stress conditions is well known^66^. We also detected differential up-regulation of genes regulating the lignin metabolism in Pokkali rice cultivar, suggesting their role in salinity stress response.

The antioxidant defense system is very effective in plants under stress conditions and allows to combat severe toxicity with the aid of several enzymes, like catalase, peroxidase and glutathione-S-transferase (GST)^67^,^68^. We found the enrichment of genes encoding enzymes, like peroxidases and GSTs, in N22 and Pokkali under desiccation and salinity stresses, respectively. Interestingly, the overexpression of GST genes has been reported to impart enhanced stress tolerance in transgenic plants^69^,^70^. The production of secondary metabolites has also been found to be crucial in stress-adaptive mechanisms in plants^22^,^23^,^71^. Phenylpropanoid biosynthesis genes have also been found to be induced by a variety of stresses^72^. We found genes involved in flavonoid derivatives and phenylpropenoid metabolism to be specifically up-regulated in N22 under desiccation stress, whereas genes involved in carotenoid and terpenoid metabolism were found to be up-regulated in Pokkali under salinity stress. The above evidences suggest that stress-adaptive responses in N22 and Pokkali rice cultivars are governed by both common and specific genes involved in various secondary metabolic pathways.

In summary, our study provides a comprehensive overview of the transcriptome of three rice cultivars with contrasting stress responses and highlights the transcriptional variations among them under control and stress conditions. Several novel transcripts and transcript isoforms specific to the rice cultivars with distinct expression patterns have been identified. IR AS has been found to be the possible mechanism responsible for the generation of transcript isoforms involved in differential abiotic stress response in the rice cultivars. In addition, we recognized some common and exclusive metabolic pathways in N22 and Pokkali cultivars, which may be important for desiccation and/or salinity stress tolerance in rice. Overall, the resource generated in this study can be used to identify most suitable candidate genes for carrying out genetic modifications in susceptible rice cultivars to generate high-yielding stress-tolerant rice cultivars.

## Methods

### Plant material and RNA isolation

The seeds of three rice (*Oryza sativa* L.) cultivars, IR64, N22 and Pokkali, were surface sterilized with 2% sodium hypochlorite for 45 min and grown on reverse osmosis (RO) water saturated cotton in a culture room (14 h light/10 h dark at temperature 28 ± 1 °C) for 14 days. The seedlings were subjected to control, desiccation and salinity stress treatments. For control treatment, seedlings were kept in RO water; for desiccation (water-deficit) treatment, seedlings were kept on filter paper^10^ and for salinity treatment, seedlings were kept in 200 mM NaCl solution^73^ for 3, 6 and 12 h time points. Three independent biological replicates for each tissue sample were harvested.

### Illumina sequencing and quality control

Total RNA was isolated from each tissue sample (control, desiccation and salinity from each time points) using TRI reagent (Sigma Life Science, St. Louis, MO) according to manufacturer’s instructions. The quantity and quality of RNA samples were determined using Nanodrop 2000 (Thermo Fisher Scientific, Wilmington, DE) and Agilent 2100 Bioanalyzer (Agilent Technologies, Singapore) as described previously^74^. Total RNA (~5 μg) (pooled in equal quantity from three biological replicates of all time points) for each genotype and condition was used for library preparation and sequencing. Libraries were prepared using Illumina TrueSeq RNA library method according to TrueSeq RNA Sample Preparation guide (Illumina Technologies, San Diego, CA) and paired-end sequencing was done using Illumina Genome Analyzer II (Illumina) to obtain reads of 100 bp length. The sequencing reads were filtered using NGSQC Toolkit^28^ at default parameters for removing low-quality (-cutOffReadLen4HQ = 70) and primer/adapter contaminated reads. The HQ reads after quality filtering were used for downstream analysis. The sequencing data generated in this study have been deposited in Gene Expression Omnibus (GEO) database under the series accession number GSE60287.

### Reference-based assembly and identification of novel transcripts

TopHat (version 2.1.0; http://tophat.cbcb.umd.edu/) and Cufflinks (version 2.2.1; http://cufflinks.cbcb.umd.edu/) softwares were used for reference-based assembly^75^. TopHat was used for mapping the HQ reads using the options–mate-inner-dist 350, –mate-std-dev 100, on the rice genome downloaded from RGAP (MSU version 7, http://rice.plantbiology.msu.edu/). Assembly was performed *via* Cufflinks using the TopHat mapping files with default parameters. The final assembly was obtained by merging the individual assemblies with default options using Cuffmerge. Functional annotation of the assembled transcripts was carried out using blastx search with an *E*-value cut-off of ≤1e-5 against the rice proteome sequence downloaded from RGAP and non-redundant (NR) protein database (downloaded on 30/04/2015, ftp://ftp.ncbi.nlm.nih.gov/blast/db/) from NCBI. The novel transcripts/transcript isoforms were identified using Cuffcompare with default parameters. Further, the annotation of novel genes/transcripts was carried out using blastx search against the NR database. We filtered the output using an *E*-value cut-off of ≤1e-5 to assign a putative function to transcripts.

### Alternative splicing analysis

Alternative splicing event determination was carried out using AStalavista web tool (version 3; http://genome.crg.es/astalavista/) at default parameters. The .gtf file from each assembly was used as input. The output provided for all the AS events from whole transcriptome data was further analyzed manually.

### Differential gene expression

Differential gene expression was determined by Cuffdiff utility provided in Cufflinks package using options, –upper-quartile-norm, –total-hits-norm and –frag-bias-correct. The transcripts with log_2_ fold change ≥1 (up-regulated genes) and ≤(−1) (down-regulated genes) with *P*-value cut off of ≤0.05 were considered as significantly differentially expressed transcripts.

### Gene ontology enrichment and pathway analysis

Gene ontology enrichment for various differentially expressed transcripts was performed using BiNGO plug-in (version 3.0.3) at Cytoscape platform (version 3.2.1; http://www.cytoscape.org/). Rice GO information for biological process and molecular function was used for gene ontology enrichment analysis. We considered a *P*-value cut-off of ≤0.05 as significant and applied hypergeometric test to identify enriched GO terms in BiNGO. Pathway analysis of differentially expressed transcripts was carried out using Mapman (version 3.5.1; http://mapman.gabipd.org/web/guest) with *P*-value cut-off of ≤ 0.05. The differentially expressed transcripts were mapped on Arabidopsis pathway genes to identify the transcripts involved in specific pathways.

### Quantitative PCR analysis

Validation of RNA-Seq data was carried out using the qRT-PCR analysis. Primers specific to selected transcripts were designed using Primer Express (version 3.0) software (Applied Biosystems, Foster City, CA) and qRT-PCR was carried out in 7500 Sequence Detection System (Applied Biosystems) as described previously^72^. The qRT-PCR was performed using at least two independent biological replicates and three technical replicates of each biological replicate for each cDNA sample. Rice ubiquitin gene (*UBQ5*) was used as a suitable internal control gene as reported in various previous studies^21^,^76^,^77^,^78^,^79^. Expression patterns of transcripts obtained from both qRT-PCR and RNA-Seq analysis were plotted in Microsoft excel 2007. Correlation coefficient was calculated using R programming package. All the primer sequences used for qRT-PCR have been provided in Supplementary Table S5.

## Additional Information

**How to cite this article**: Shankar, R. *et al*. Transcriptome analysis in different rice cultivars provides novel insights into desiccation and salinity stress responses. *Sci. Rep*. **6**, 23719; doi: 10.1038/srep23719 (2016).

## Supplementary Material

Supplementary Information

Supplementary Table S1

## Figures and Tables

**Table 1:** Summary of read data, mapping and reference-based assembly obtained for each rice cultivars.

	Raw reads			High quality reads			Mapped reads (%)			Assembly output	
	Control	Desiccation	Salinity	Control	Desiccation	Salinity	Control	Desiccation	Salinity	Total transcripts	Novel transcripts (loci)
IR64	58565758	58421624	63359274	54230024	52368210	56489534	95.5	94.2	95.4	88652	2499 (1832)
N22	96289178	71160532	74711050	81129568	63332500	68019518	95.0	93.9	95.0	90556	3054 (2265)
Pokkali	62150476	59917678	61226268	54962644	54889310	56031966	95.6	95.1	95.2	87887	2433 (1785)

**Table 2:** Highly enriched gene ontology (GO) terms for the differentially expressed intron retention (IR) transcripts under desiccation and salinity stresses in N22 and Pokkali rice cultivars, respectively.

GO term	*P*-value
Desiccation stress	
BP: response to abiotic stimulus	8.03E-07
BP: glycoside metabolic process	0.000158
BP: response to desiccation	0.000249
BP: disaccharide biosynthetic process	0.000296
BP: catabolic process	0.000377
BP: oligosaccharide biosynthetic process	0.000443
BP: trehalose biosynthetic process	0.000528
BP: glycoside biosynthetic process	0.000585
BP: disaccharide metabolic process	0.000585
BP: trehalose metabolic process	0.000603
MF: nucleic acid binding	1.36E-05
MF: hydrolase activity	3.97E-05
MF: catalytic activity	8.02E-05
MF: hydrogen-exporting ATPase activity	0.000357
MF: DNA binding	0.00053
CC: thylakoid	3.13E-03
Salinity stress	
BP: glycoside metabolic process	8.44E-05
BP: disaccharide metabolic process	0.000138
BP: carbohydrate metabolic process	0.000144
BP: di-, tri-valent inorganic cation transport	0.000234
BP: oligosaccharide metabolic process	0.000241
BP: trehalose metabolic process	0.000459
BP: cellular carbohydrate metabolic process	0.000671
BP: calcium ion transmembrane transport	0.000691
MF: catalytic activity	6.19E-05

All the differentially expressed transcripts produced through IR event under desiccation and salinity stresses were subjected to gene ontology (GO) enrichment analysis. For the enrichment, *P*-value cut-off was <0.001. CC: Cellular component, BP: Biological process and MF: Molecular function.
