# Supplementary material for: Transcriptome analysis in different rice cultivars provides novel insights into desiccation and salinity stress responses
Source: Sci Rep. 2016 Mar 31;6:23719. doi: 10.1038/srep23719 (PMC4814823; doi:10.1038/srep23719)
Supplement: Supplementary Information [file srep23719-s1.pdf]

Supplementary information

Transcriptome analysis in different rice cultivars provides novel insights into desiccation and salinity stress responses

Rama Shankar[1], Annapurna Bhattacharjee[1] and Mukesh Jain[1,2]*

[1]Functional and Applied Genomics Laboratory, National Institute of Plant Genome Research (NIPGR), Aruna Asaf Ali Marg, New Delhi - 110067, India.
[2]School of Computational and Integrative Sciences, Jawaharlal Nehru University, New Delhi-110067, India

*Corresponding author

(a) IR64-Ct

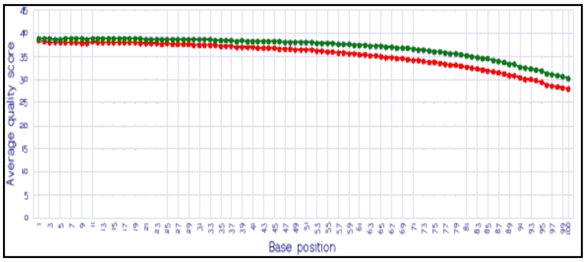

paired-end 1

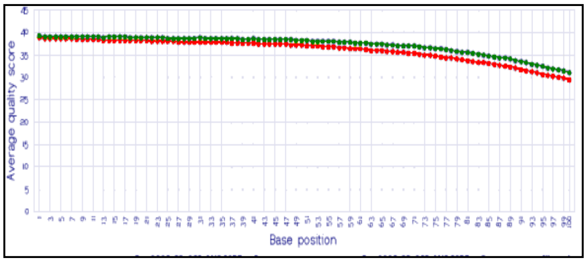

paired-end 2

(b) IR64-Ds

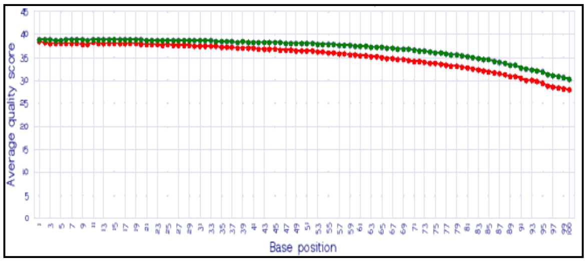

paired-end 1

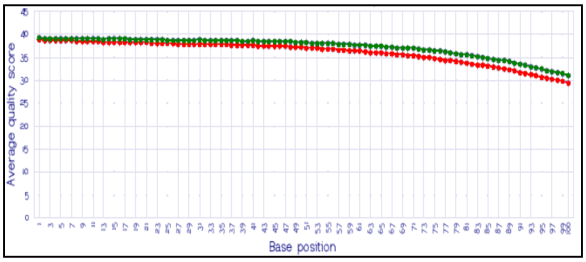

paired-end 2

(c) IR64-Ss

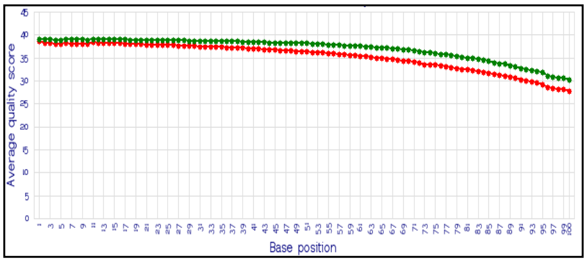

paired-end 1

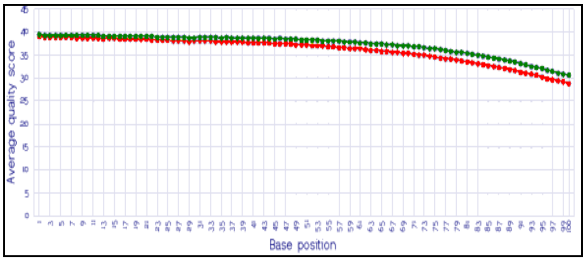

paired-end 2

(d) N22-Ct

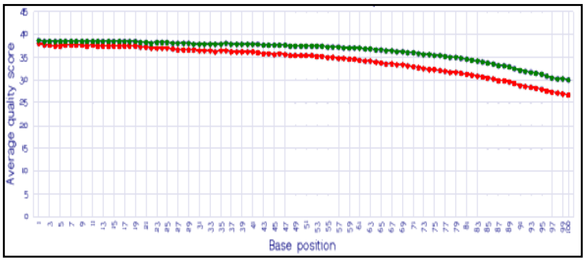

paired-end 1

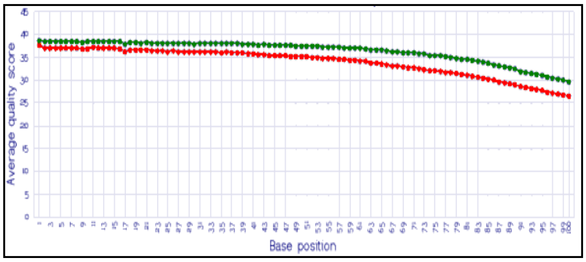

paired-end 2

(e) N22-Ds

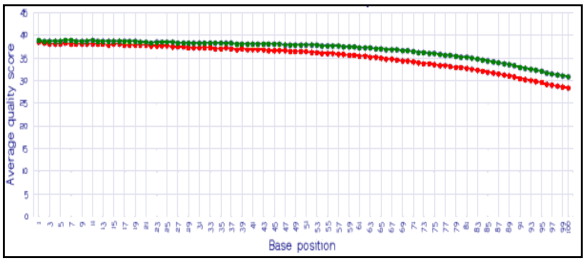

paired-end 1

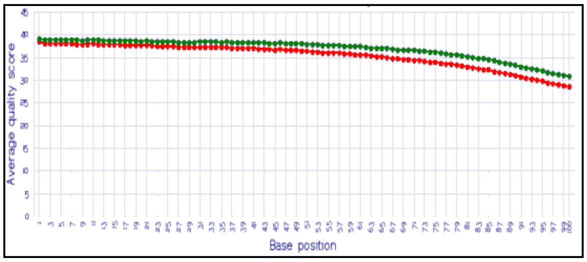

paired-end 2

(f) N22-Ss

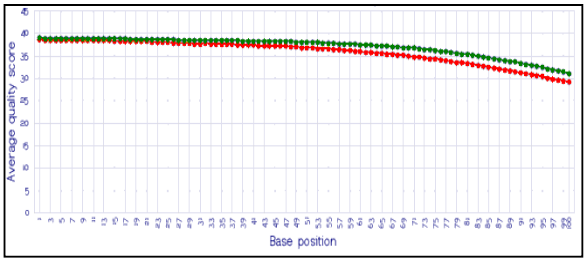

paired-end 1

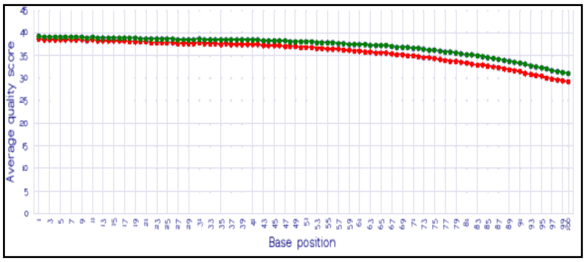

paired-end 2

(g) Pokkali-Ct

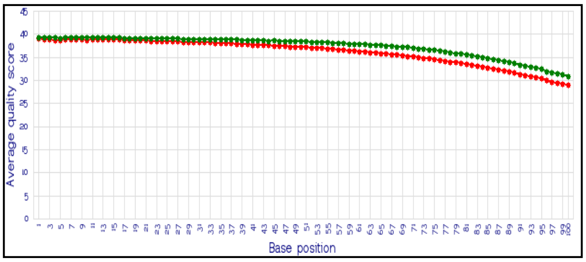

paired-end 1

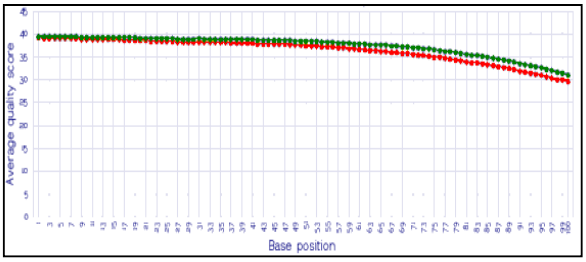

paired-end 2

(h) Pokkali-Ds

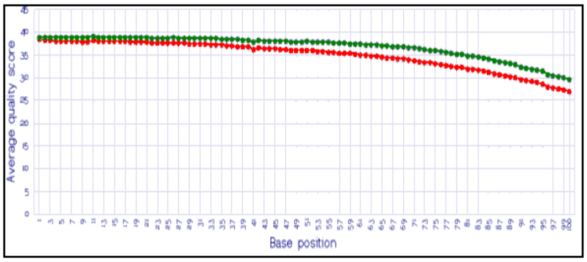

paired-end 1

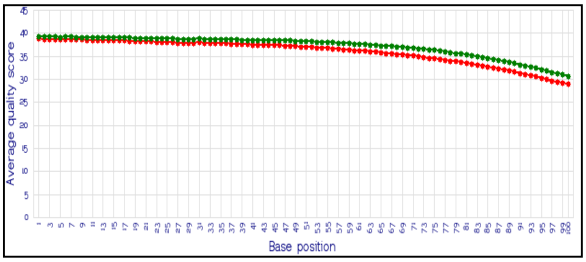

paired-end 2

(g) Pokkali-Ss

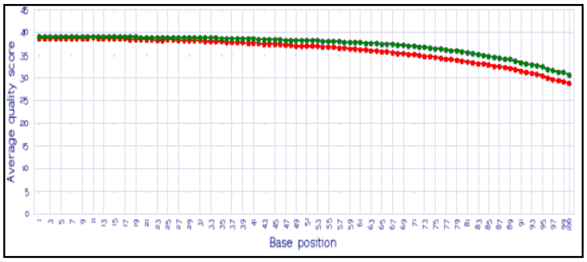

paired-end 1

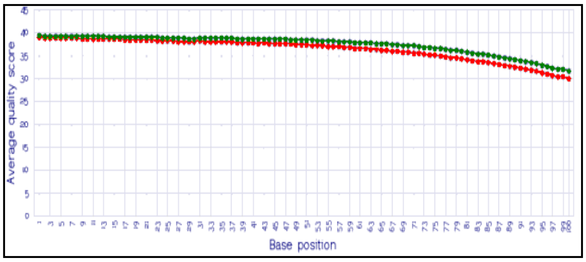

paired-end 2

Fig. S1. Average Phred quality scores of each base position for unfiltered and filtered reads for all the sequencing samples (a) to (g). The red line represents Phred score for unfiltered reads and the green line represents Phred score for filtered reads. These diagrams showing the improvement in quality of reads after filtering. Ct-Control, Ds- Desiccation stress and Ss-salinity stress.

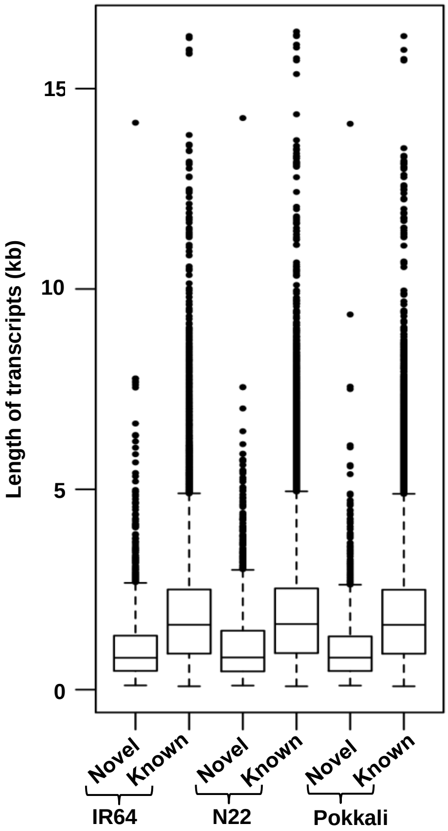

Fig. S2. Length distribution of the assembled novel transcripts and known transcripts in IR64, N22 and Pokkali rice cultivars.

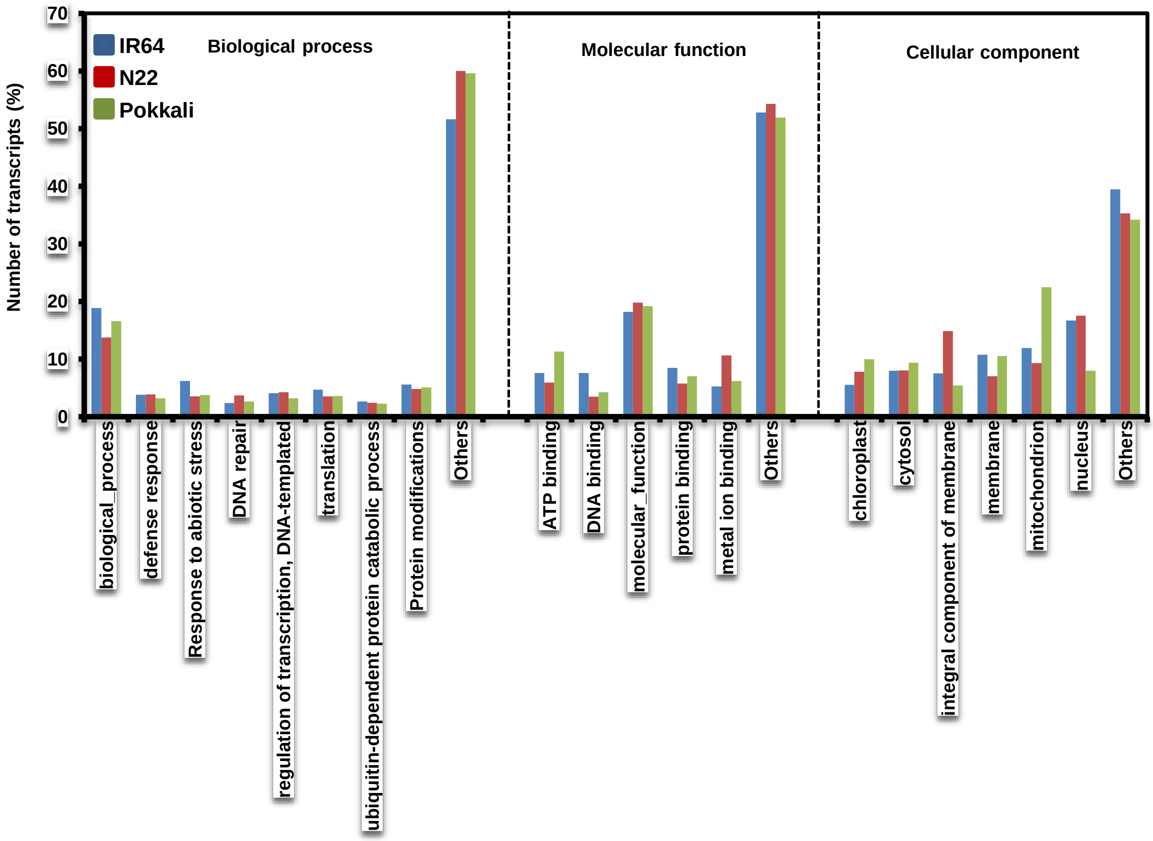

Fig. S3. GOSlim term assignment to novel transcripts identified in IR64, N22 and Pokkali rice cultivars.

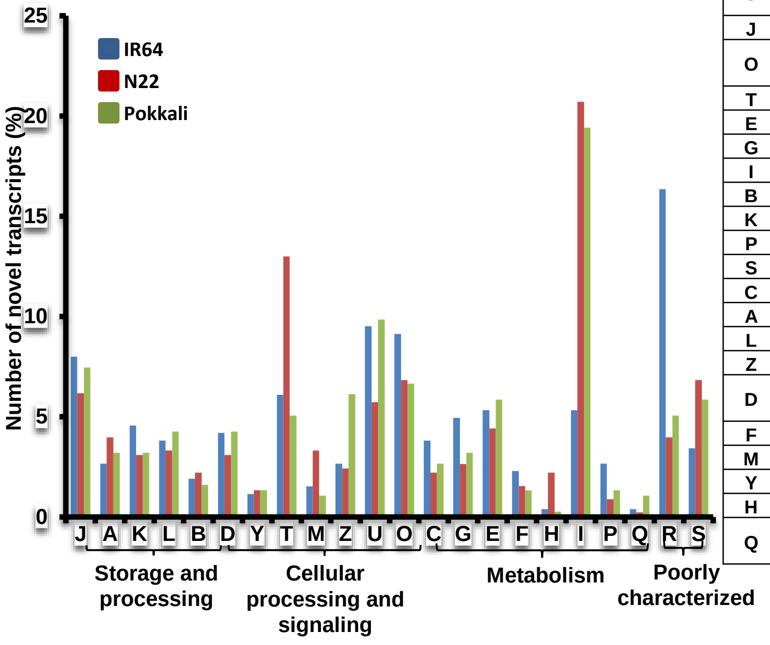

KOG code	KOG description
R	General function prediction only
U	Intracellular trafficking, secretion, and vesicular transport
J	Translation, ribosomal structure and biogenesis
O	Posttranslational modification, protein turnover, chaperones
T	Signal transduction mechanisms
E	Amino acid transport and metabolism
G	Carbohydrate transport and metabolism
I	Lipid transport and metabolism
B	Chromatin structure and dynamics
K	Transcription
P	Inorganic ion transport and metabolism
S	Function unknown
C	Energy production and conversion
A	RNA processing and modification
L	Replication, recombination and repair
Z	Cytoskeleton
D	Cell cycle control, cell division, chromosome partitioning
F	Nucleotide transport and metabolism
M	Cell wall/membrane/envelope biogenesis
Y	Nuclear structure
H	Coenzyme transport and metabolism
Q	Secondary metabolites biosynthesis, transport and catabolism

Fig. S4. KOG analysis of the novel transcripts identified in IR64, N22 and Pokkali rice cultivars.

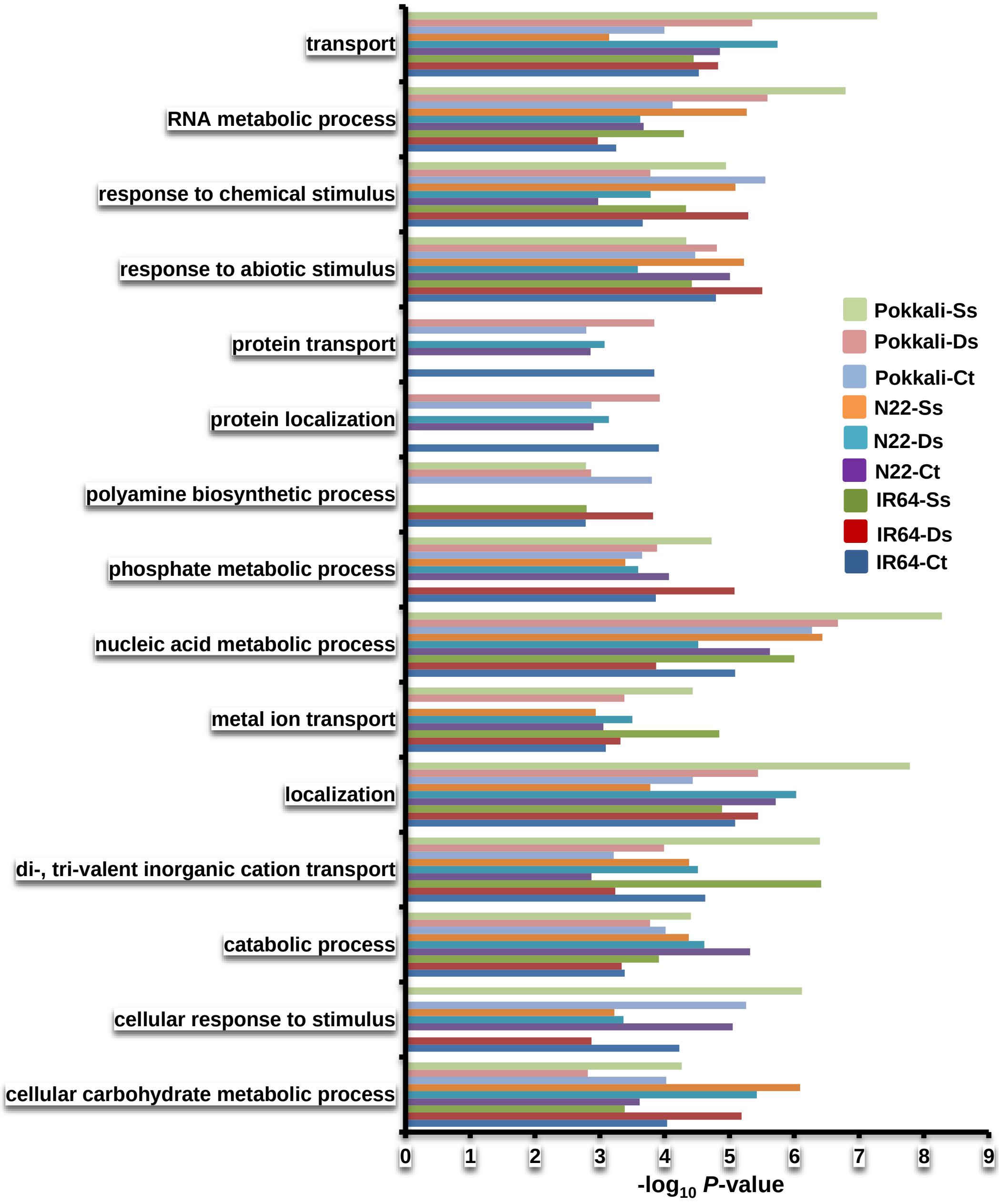

Fig. S5. Gene ontology (GO) enrichment for biological process category of transcripts produced through intron retention alternative splicing events in all the three rice cultivars under control and stress conditions. The GO enrichment score was obtained by BiNGO software as $-\text{Log}_{10}$ *P*-values (*P*<0.02). Ct-control condition, Ds-desiccation stress and Ss-salinity stress.

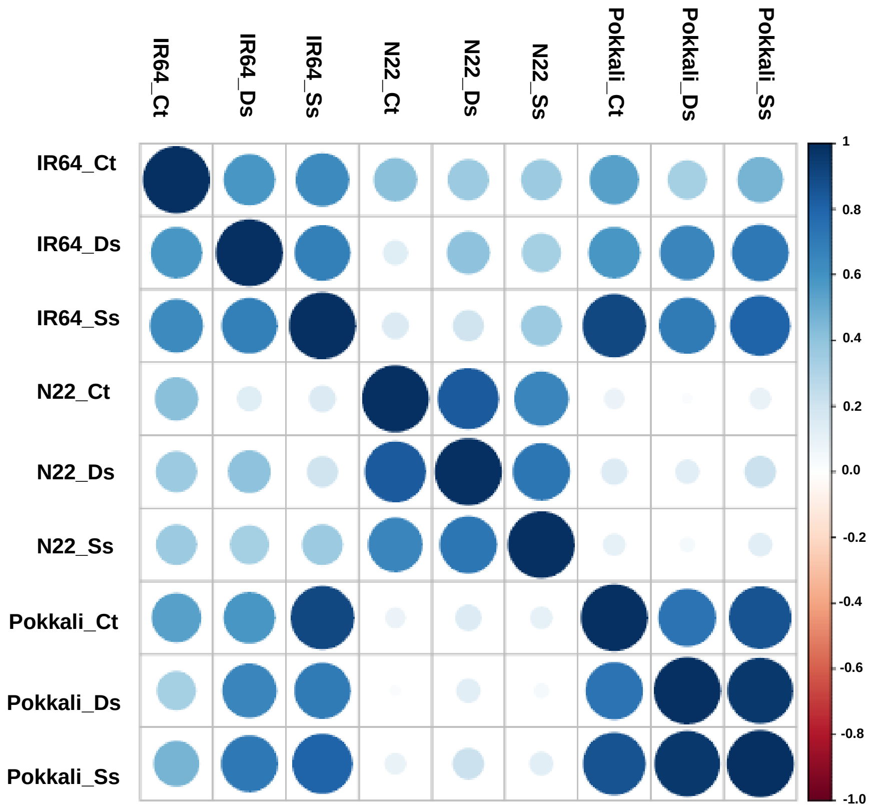

Fig. S6. Correlation of expression values in all the three rice cultivars under control and stress conditions. The color coding represents the correlation coefficient. Ct- Control, Ds-desiccation stress and Ss-salinity stress.

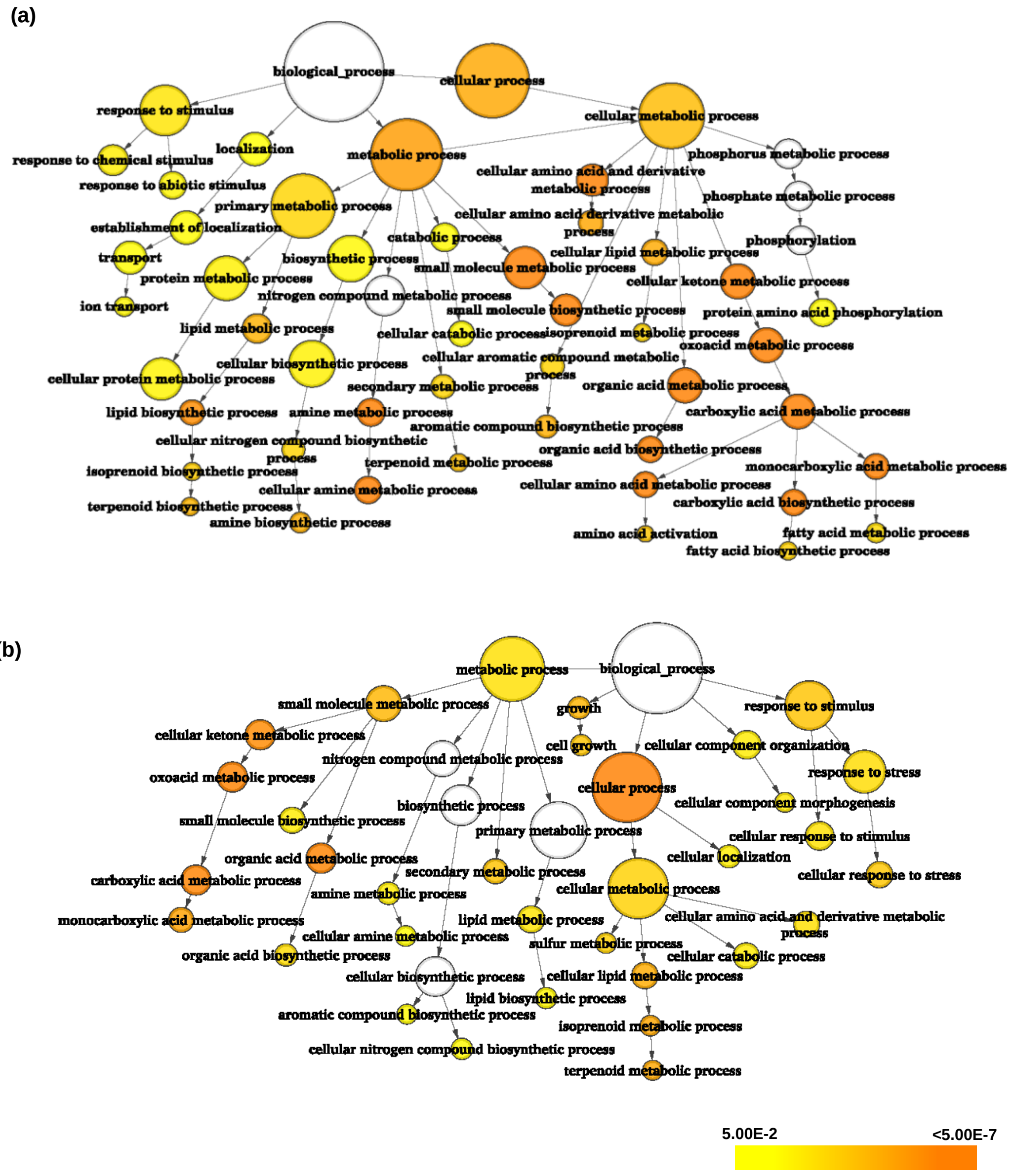

Fig. S7. Gene ontology (GO) enrichment analysis of differentially expressed transcripts in rice cultivars under control condition. GO enrichment for differentially expressed transcripts specific to N22 (a) and Pokkali (b) rice cultivars under control condition is shown. Node size is proportional to the number of transcripts in that category and color shaded is according to significance level (white represents-No significant difference, color scale yellow-*P*-value=0.05, orange- P-value<0.0000005).

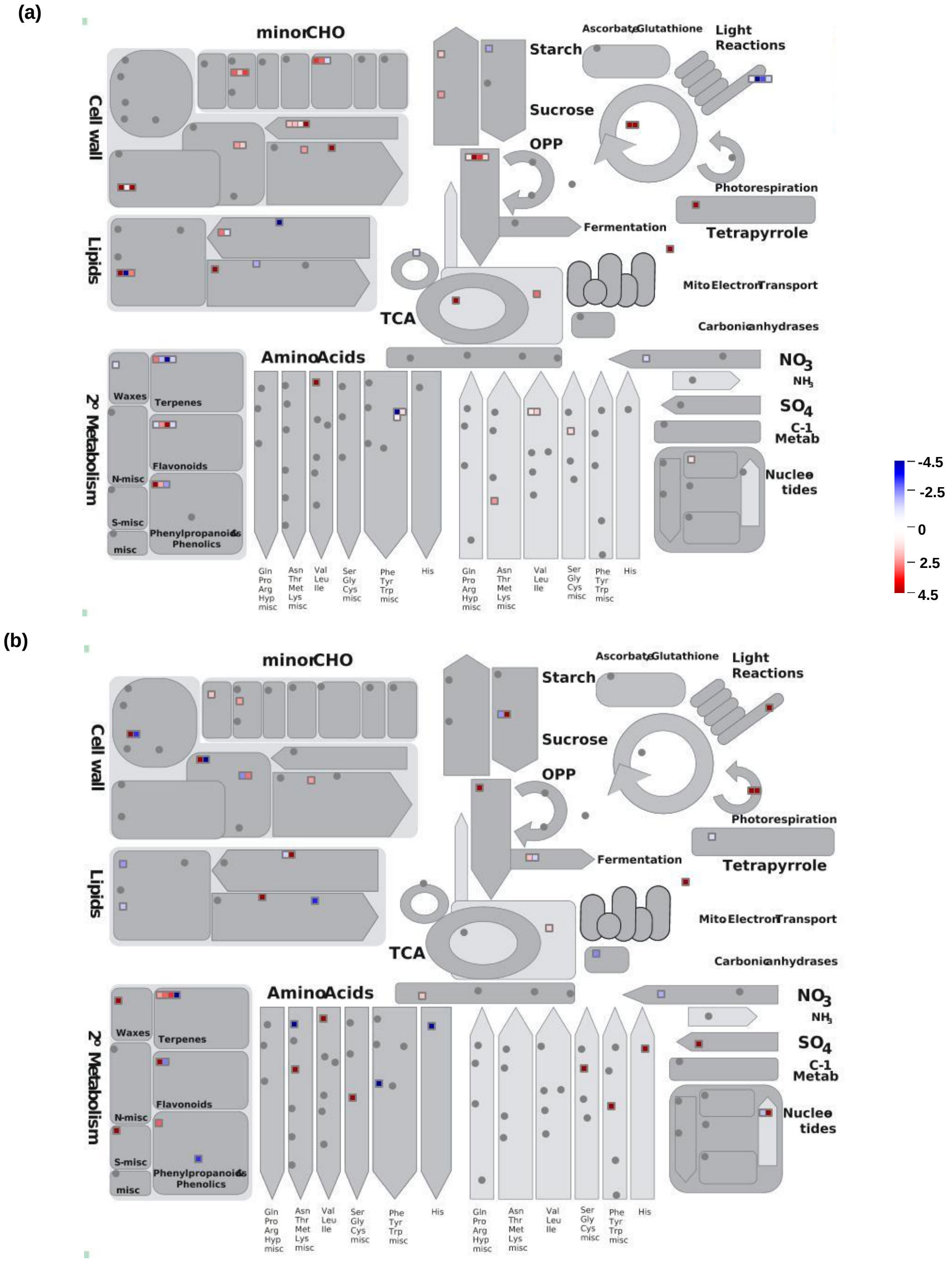

Fig. S8. Overview of metabolic pathways in rice cultivars under different stresses. Metabolic pathways overview of differentially expressed transcripts specific to N22 rice cultivar under desiccation stress (a) and differentially expressed transcripts specific to Pokkali rice cultivar under salinity stress (b) are shown. Color coding, red, up-regulated transcripts and blue, down-regulated transcripts.

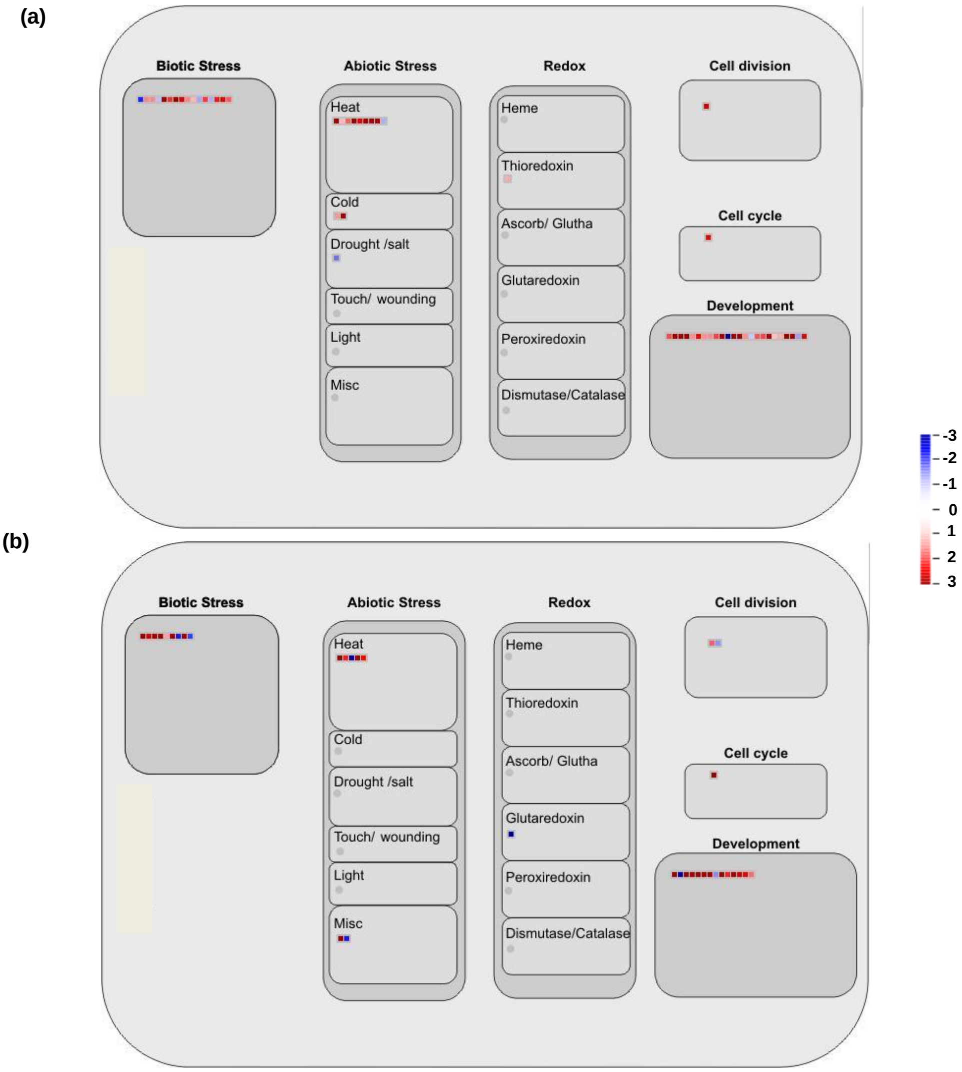

Fig. S9. Cellular overview of differentially expressed transcripts in rice cultivars under stress conditions. The differentially expressed transcripts enriched for various cellular functions in N22 under desiccation stress (a) and in Pokkali under salinity stress (b) are shown. Red, up-regulated transcripts and blue, down-regulated transcripts.

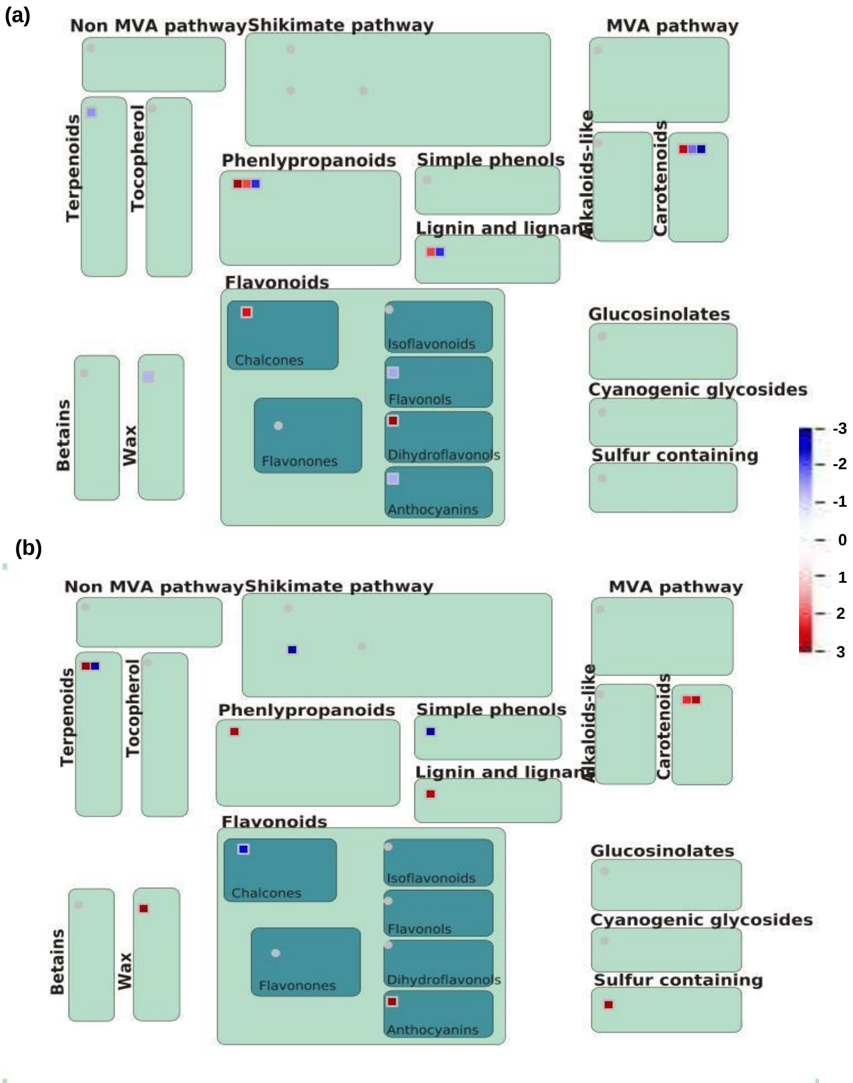

Fig. S10. Secondary metabolic pathways overview of differentially expressed transcripts in rice cultivars under different stresses. Various secondary metabolic pathways were enriched in N22 rice cultivar under desiccation stress (a) and Pokkali rice cultivar under salinity stress (b). Red, up-regulated transcripts and blue, down-regulated transcripts.

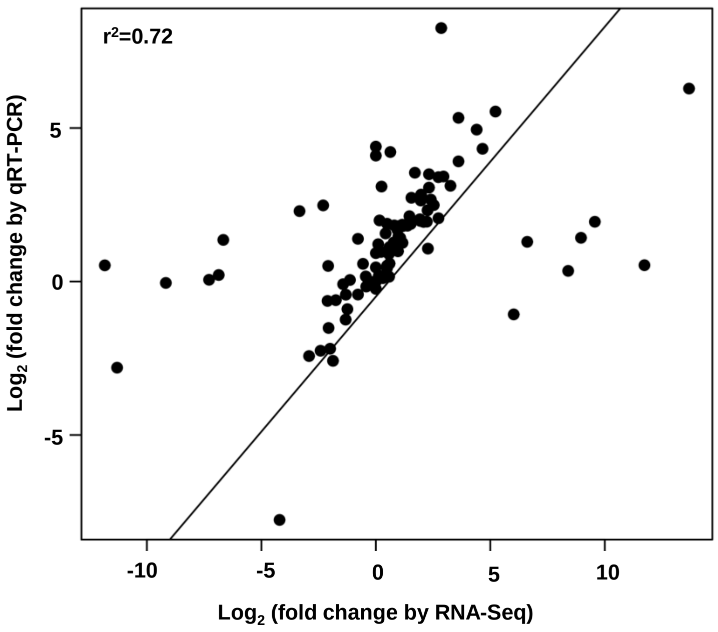

Fig. S11. Correlation plot representing expression values of the transcripts obtained from qRT-PCR and RNA-seq data analysis.

Table S1. Assembly statistics of individual conditions (control, desiccation and salinity stress) for each rice cultivars, namely IR64, N22 and Pokkali.

**Parameters**	**IR64**	**N22**	**Pokkali**
Total transcripts	88652	90556	87887
Transcripts with length >1000 bp	62767	64378	62087
Min transcript length (bp)	102	102	102
Average transcript length (bp)	1845	1864	1838
N50 transcript length	2444	2467	2433

Table S2. Number of AS events in all the three rice cultivars (IR64, N22 and Pokkali) under control, desiccation and salinity stress conditions.

AS events	IR64			N22			Pokkali		
	Ct	Ds	Ss	Ct	Ds	Ss	Ct	Ds	Ss
IR	7922	7631	7768	8810	8254	8510	7579	7689	7851
AA	4657	4407	4596	4878	4587	4732	4611	4445	4757
AD	2494	2333	2486	2588	2471	2538	2487	2389	2497
ES	1772	1865	1880	1818	1860	1971	1816	1856	1909
IR1 or IR2	382	365	354	449	417	417	319	371	353
IR1+IR2	1056	980	991	1194	1137	1125	966	994	991
AA+IR1	191	187	195	211	192	216	190	182	184
AA+IR2	192	168	180	196	179	205	164	182	180
AD or AA	271	289	304	321	296	298	329	279	310
ES1+ES2	209	208	213	186	209	228	171	210	210
Others	2086	2047	2112	2400	2244	2314	2093	2062	2076

Ct- control condition, Ds-desiccation stress, Ss-salinity stress, IR-intron retention, AA-alternate-3'acceptor, AD-alternative-5'donor, ES-exon skipping.

Table S3. List of all the differentially expressed transcripts under control and stress conditions in the rice cultivars (*Available as a separate MS Excel file*).

Table S4. Differentially expressed transcripts under desiccation and salinity stresses produced through various AS events in IR64, N22 and Pokkali rice cultivars.

AS events	IR64		N22		Pokkali	
	Desiccation	Salinity	Desiccation	Salinity	Desiccation	Salinity
**IR**	527	135	427	315	548	148
**AA**	409	96	332	250	429	120
**AD**	225	63	177	129	230	69
**ES**	170	41	122	93	170	51
**IR1+IR2**	62	19	56	38	59	22
**IR1 or IR2**	30	12	26	15	28	6
**AD or AA**	29	14	20	10	21	12
**AA+IR1**	22	6	18	14	20	4
**AA+IR2**	14	3	11	8	16	6
**ES1+ES2**	21	4	13	13	22	6
**Others**	37	9	30	22	35	13

IR-intron retention, AA-alternate 3'acceptor, AD-alternative 5'donor, ES-exon skipping.

Table S5. List of primers used for qRT-PCR analysis.

S. no.	Gene identifier	Primer sequence
1	LOC_Os04g43680	F-CAAGAGGAGCAGAGCAGTTCAG
		R-GCCCTCCCCATTGTGCTT
2	LOC_Os01g03520	F-GCTTAGCCAAACAATCAGAATCAG
		R-CATTGTCCTGTGATCCCTCCTT
3	LOC_Os02g08440	F-CAAGGATTGACGATTGCTACTGA
		R-CCCGCAGTTGCAAGAAGAAG
4	LOC_Os02g42810	F-GCCGAGAAAAGGGTTGCA
		R-TGCATATCTCGAGCCCAAGTC
5	XLOC_006923	F-CCATCAGGAGCGAAGCAAAT
		R-TGCTTCCTTGATGGCAAGTCT
6	LOC_Os01g53220	F-TCCTCCTCCCCTCCTACTTCA
		R-GCGGAATCCGTAGGTGTTGA
7	LOC_Os01g72530	F-GGCTCCGACCAGGACATC
		R-TGAGATCGAACCTGCAGATCA
8	XLOC_013454	F-GCTGTCTTAGTCTCCTTTATTATCTTATGCA
		R-GTAAGCTCATAGAAGCATCAACACAAC
9	LOC_Os11g03300	F-GCCGAGGTCAACATCTACAAGTG
		R-CGCCGAACAAAGCCTTACC
10	LOC_Os01g72370	F-CCTACTACTACTGGTCTGGCTTGGT
		R-CCCGCTTCCGTCTCAAAAG
11	LOC_Os11g31640	F-TCCAGGGAGTGCCTTAGACAA
		R-AGGTGTGGCAGGGAATGC
12	LOC_Os11g20160	F-TGGTGACGCGTTCCAGTGTA
		R-GCAGAACAAGCTTGAGCATGACT
13	LOC_Os01g72530	F-GGCTCCGACCAGGACATC
		R-TGAGATCGAACCTGCAGATCA
14	LOC_Os01g53220	F-TCCTCCTCCCCTCCTACTTCA
		R-GCGGAATCCGTAGGTGTTGA
15	UBQ5 (internal control)	F-ACCACTTCGACCGCCACTACT
		R-ACGCCTAAGCCTGCTGGTT
